# Novel Biorefinery Approach for Phycocyanin Extraction and Purification and Biocrude Production from *Arthrospira platensis*

**DOI:** 10.1021/acs.iecr.2c03683

**Published:** 2023-03-08

**Authors:** Jennifer Sánchez-Laso, Juan J. Espada, Rosalía Rodríguez, Gemma Vicente, Luis Fernando Bautista

**Affiliations:** † Department of Chemical and Environmental Technology, ESCET, Universidad Rey Juan Carlos, Móstoles, 28933 Madrid, Spain; ‡ Department of Chemical, Energy and Mechanical Technology, ESCET, Universidad Rey Juan Carlos, Móstoles, 28933 Madrid, Spain

## Abstract

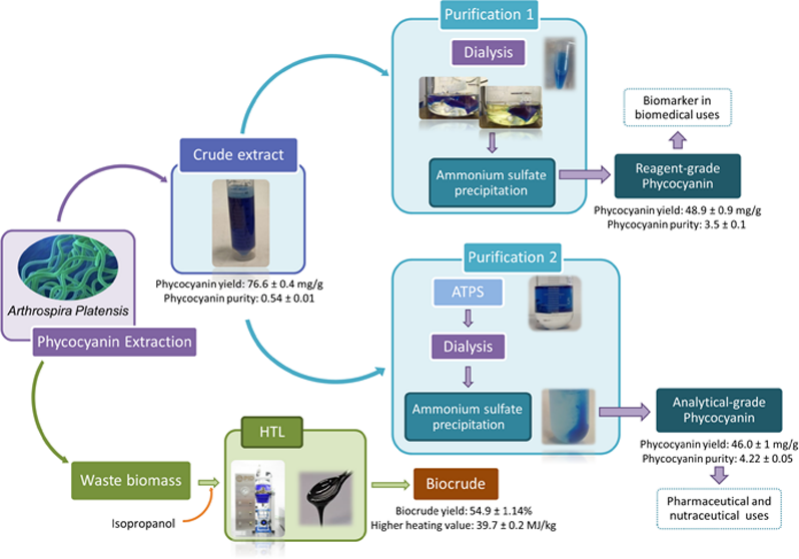

A new biorefinery from *Arthrospira platensis* was proposed to obtain phycocyanin (PC) and a biocrude by hydrothermal liquefaction (HTL). PC is a high-added-value phycobiliprotein widely used as a food colorant and in the nutraceutical and pharmaceutical industries. However, the use of conventional solvents in the extraction process and the purity grade of the extract are shortcomings in bioproduct production. PC was extracted using a reusable ionic liquid [EMIM][EtSO_4_], achieving a PC purity of the lowest commercial grade. Therefore, two downstream processes were applied: (1) dialysis + precipitation and (2) aqueous two-phase system (ATPS) + dialysis + precipitation. After the second purification process, the PC purity increased remarkably to reach the analytical grade for pharmaceutical and nutraceutical applications. The waste biomass (WB) obtained in the PC extraction was valorized by hydrothermal liquefaction (HTL) to produce a biocrude. The biocrude yield and composition remarkably enhanced using isopropanol at 350 °C as a cosolvent.

## 1 Introduction

Microalgae and cyanobacteria can accumulate bioproducts, such as pigments (carotenoids, phycobiliproteins, and chlorophylls), polyunsaturated fatty acids (PUFA), sterols, vitamins, and polysaccharides with applications in human health, cosmetic, pharmaceutical, and nutraceutical industries.^1−7^ Besides, it is well known that microalgae and cyanobacteria produce biofuels (biodiesel, bioethanol, biocrude, biogas).^8−11^ Therefore, their versatility makes them an interesting feed for a biorefinery to obtain bioproducts and biofuels.

Phycobiliproteins (phycocyanin, allophycocyanin, and phycoerythrin) are high-value bioproducts commonly presented in cyanobacteria. These water-soluble and brilliant-colored proteins capture light energy by acting as photosynthetic pigments. They have potential applications in the nutraceutical, pharmaceutical, food, and cosmetic industries.^6,12,13^ Particularly, phycocyanin (PC) is a natural blue pigment, mainly present in the cyanobacteria *Arthrospira platensis* (with contents up to 13%, dry weight basis).^7,14^ PC is highly demanded due to its pharmacological properties, such as its antioxidant and anti-inflammatory effects, potential use as a food colorant for chewing gum and sweets, and as an active cosmetic ingredient. PC will have an estimated global market value of US$245.5 million by 2027.^15^ This bioproduct is commercialized according to its purity grade. Thus, it is divided into four grades regarding its potential commercial applications.^16^ The cost of food-grade PC (purity > 0.7) is around 0.13 US$ mg^–1^, whereas the price of analytical grade (purity > 4.0) can be as high as 33 US$ mg^–1^.^17^

Ionic liquids (ILs) have emerged as a promising alternative for the efficient extraction of PC,^18^ as they are green solvents that show unique properties, such as high thermal and chemical stability, strong solubility, solvating power, and the possibility to be reused. [Emim][EtSO_4_] has been applied in the PC extraction of *A. platensis* with successful results in terms of extraction yield and IL recovery through a dialysis process.^14^ In addition, food-grade PC was obtained without an additional purification process. Therefore, further purification stages would provide even higher-grade PC.

The selection of appropriate purification methods, using a minimum number of scalable steps, is essential for the commercial development of PC. The purification downstream process involves precipitation, centrifugation, ultrafiltration, dialysis, a biphasic aqueous system, or a sequence of these steps. Aqueous two-phase system (ATPS) shows several advantages, such as energy efficiency, short separation times, and ease of operation.^19^ ATPS is a mixture of water and two other compounds: a polymer and a salt.^20^ The separation of the phases can be adjusted by modifying the composition of the above compounds, which allows their distribution in each phase.^21,22^ Furthermore, ATPS allows the suitable use of ILs for PC purification.

The PC extraction of *A. platensis* using an IL also produces residual biomass with high amounts of lipids and carbohydrates,^14^ which requires further exploitation within a circular economy context. The residual biomass after the extraction step could be valorized by hydrothermal liquefaction (HTL) to obtain a biocrude that could then be processed in a refinery to obtain different biofuels, such as jet fuel. HTL is a well-known thermochemical process that can work directly with wet biomass at high temperatures and pressure, thus avoiding the drying process, which is highly energy-consuming.^23,24^ An increase in the biocrude yield has been reported with alcohols as cosolvents in microalga HTL.^25^ They act as an extracting agent improving the conversion of lipids into liquid fuels, which would be advantageous in the waste biomass HTL resulting in the PC extraction.

**Figure 1:**
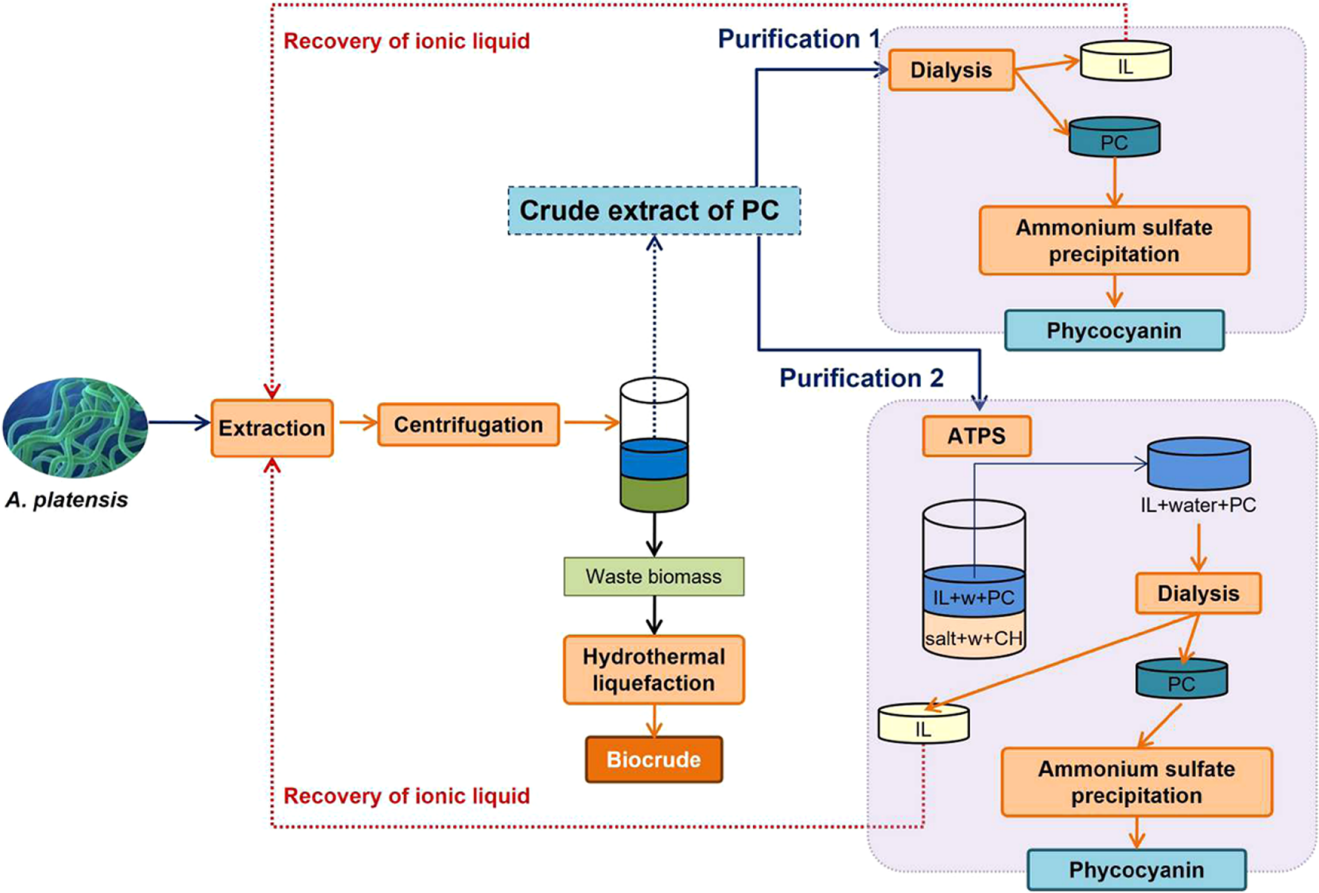
Scheme of *A. platensis* biorefinery. It includes phycocyanin extraction and purification procedures (purification 1: dialysis + precipitation or purification 2: ATPS + dialysis + precipitation) and waste biomass used to obtain biocrude by HTL.

This work proposes a biorefinery scheme for PC and biocrude production from *A. platensis* (Figure 1). The proposed scheme is in line with the recent attempts to integrate the microalga bioproduct recovery with the production of several products to enhance the profitability of the overall processes.^26^ Most biorefinery schemes have been focused on lipid extraction and further biodiesel production.^27,28^ Biorefinery schemes are scarce for the rest of the bioproducts (proteins, carbohydrates, pigments). Regarding pigments, Senatore et al. have reported an integrated biorefinery-based procedure to obtain phycobiliproteins, poly(hydroxybutyrate) (PHB), and lipids.^29^ Likewise, Martins et al. have reported the extraction and fractionation of pigments such as chlorophyll and fucoxanthin from *Saccharina latissima*, using a phosphonium-based IL surfactant combined with sunflower oil and water.^30^ However, the residual biomass after pigment extraction was not used in both cases. The novelty of our work is the use of the same IL to carry out both extraction and purification steps to produce PC, together with the further use of the resulting waste biomass to obtain a biocrude. This novel biorefinery approach can be of interest concerning the use of an IL and the combined production of bioproducts (phycobiliproteins) and biofuels (bio-oil) using microalgae or cyanobacteria as raw material.

According to the biorefinery shown in Figure 1, PC extraction was carried out with [Emim][EtSO_4_] IL and, for further purification, two approaches were tested: (1) single dialysis + precipitation (purification approach 1) and (2) ATPS + dialysis + precipitation (purification approach 2). The waste biomass resulting from the extraction step was processed by HTL, evaluating the effect of temperature and type of alcohol, as cosolvent, on the biocrude yield and quality.

## 2 Experimental Section

### 2.1 Biomass Characterization

*A. platensis*, characterized in our previous work,^14^ was used as biomass. Table 1 summarizes the biochemical composition and elemental analysis of *A. platensis*.

**Table 1:** Characterization of *A. platensis* (Dry Weight Basis)

biochemical composition (wt %)		elemental composition (wt %)	
total proteins^a^	65.40 ± 1.80	C	47.20 ± 0.20
phycocyanin	8.84 ± 0.06	H	6.59 ± 0.07
allophycocyanin	3.25 ± 0.08	N	11.00 ± 0.10
phycoerythrin	1.69 ± 0.03	S	0.33 ± 0.03
lipids	11.20 ± 0.80	O	22.10 ± 0.40
total carbohydrates	19.40 ± 1.30		
solubles	1.34 ± 0.01		
ash	5.40 ± 0.15		

a Include phycobiliproteins.

### 2.2 Phycocyanin Extraction

The IL 1-ethyl-3-methylimidazolium ethyl sulfate, [Emim][EtSO_4_], (Sigma-Aldrich. St. Louis, Missouri) was used for the extraction step following the method reported by Sánchez-Laso et al.^14^ For this purpose, 0.18 g of the cyanobacterium biomass was mixed with 10 mL of a 20.86 wt % aqueous solution of [Emim][EtSO_4_]. The mixture was stirred for 30 s in a vortex mixer and then sonicated for 25 min at room temperature in an Elmasonic P ultrasound bath (Elma Schmidbauer GmbH. Singen, Germany) at a constant frequency (37 kHz) and amplitude (80%, 656 W).

Then, the blend was centrifuged at 10 000 rpm (12 857*g*-force) for 10 min in an Eppendorf Centrifuge 5910 (Eppendorf. Hamburg, Germany) to separate the supernatant (crude extract of PC) from the waste biomass. After centrifugation, the pellet was subjected to two consecutive washing steps: adding 0.5 mL of deionized water and further centrifuging. That procedure removed the small amount of IL remaining after the first centrifugation step, and no traces of IL were detected. The extracted yield of PC from the dry biomass (*E*_PC_) was measured by spectrophotometric absorption in a UV–Vis-Nanodrop 1000 (Thermo Fisher Scientific. Wilmington, Delaware) using eq 1, adapted from previous studies.^31^ The purity of PC (*P*_PC_) was calculated as a fraction of the total protein content by using eq 2(32)
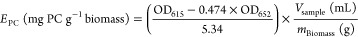
1
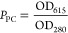
2where *V*_sample_ is the volume (mL) of the sample, *m*_Biomass_ is the mass (g) of dry microalgae, and OD_615_, OD_652_, and OD_280_ are the optical density values at 615, 652, and 280 nm, respectively.

### 2.3 Phycocyanin Purification

PC purification was carried out using two approaches for the PC crude extract: (1) dialysis + precipitation and (2) ATPS + dialysis + precipitation.

#### 2.3.1 Purification Approach 1: Dialysis + Precipitation

The dialysis-based process reported by Sánchez-Laso et al.^14^ was previously used for IL recovery in the PC extraction stage. This procedure was carried out using a membrane with a molecular weight cutoff of 14 kDa (Sigma-Aldrich). After conditioning the membrane, the PC crude extract was introduced, and the set was immersed in deionized water (volume ratio 1:4) for 4 h under continuous stirring. Then, the loaded membrane was placed into fresh deionized water, and the operation was repeated three times.

The dialyzed process was followed by a precipitation step with ammonium sulfate, which shows many advantages compared to other precipitating agents since it prevents protein denaturalization due to its low heat of solubilization and bacteriostatic effect.^33,34^ PC precipitation was performed at room temperature in two steps (i.e., 0–20 and 20–50% of saturation). Ammonium sulfate was added until a 20% saturation was attained under continuous stirring. The mixture was kept in constant stirring for 1 h at room temperature. Then, agitation was turned off, and the solution was maintained overnight and centrifuged. The supernatant was recovered, and ammonium sulfate was added again up to 50% saturation, repeating the process.^35^ The resulting precipitate, rich in PC, was dissolved in 10 mL of sodium phosphate buffer pH 7.0. The spectrophotometric absorbance of the solution was measured to determine the PC purity according to eq 2.

The purification stage of the PC extract was assessed through the purification factor (PF), estimated as the ratio between the purity of PC in the crude extract and that in each purified fraction,^16,36,37^ according to eq 3
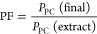
3

#### 2.3.2 Purification Approach 2: ATPS + Dialysis + Precipitation

The binodal curve describes the biphasic equilibrium of the ATPS, and the composition of each phase is determined by the corresponding tie-lines, whose construction is detailed in the Supporting Material.

The ATPS system, formed by [Emim][EtSO_4_] (IL), K_2_HPO_4_ (salt), and water, was prepared in 25 mL centrifuge glass tubes that contained the appropriate amounts of all the three components above. Before adding the crude extract into the ATPS system, the pH was adjusted to 6.5 using HCl (ACS reagent, 37%, Sigma-Aldrich) because PC is stable within the pH range 6.0–7.0 and starts degradation at pH values above 7.^38,39^ Then, the ATPS system and the crude extract were mixed in a vortex for 1 min. The blend was centrifuged at 4000 rpm for 5 min to complete the separation of both phases. The bottom phase corresponded to the salt-rich phase, while the IL-rich phase was the top. Then, the phases were separated carefully. Proteins, including PC, are preferentially concentrated in the top phase,^39,40^ whereas carbohydrates are ideally partitioned in the bottom phase. The negatively charged amino acids on the protein surface strongly interact with the cation of the ionic liquid (positively charged), promoting the transfer of proteins to the phase rich in ionic liquid.^41−43^

Both volume fractions in the ATPS system were measured, and each fraction was analyzed by spectrophotometric absorption (UV–Vis-Nanodrop 1000). The concentration of PC was calculated using eq 4, derived from eq 1
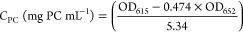
4The recovery of PC in the top and bottom phases of the ATPS system was calculated, respectively, as
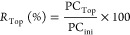
5
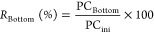
6where PC_Top_ and PC_Bottom_ represent the amount of phycocyanin (mg) in the top and bottom phases, respectively, and PC_ini_ is the amount of phycocyanin (mg) in the crude extract.

The top phase (protein-rich) was dialyzed and then treated with ammonium sulfate to precipitate and purify the PC extract, following the methods described in Section 2.3.1. PC purity and purification factor for each purified PC were determined according to eqs 2 and 3, respectively.

### 2.4 Waste Biomass Valorization

The waste biomass resulting from PC extraction was processed by HTL to produce biocrude. The process was performed with different cosolvents (methanol, ethanol, and isopropanol) to evaluate their influence on the biocrude quality and yield using a waste biomass/cosolvent ratio of 1:10 and a water/alcohol ratio of 1:1. As a control, the HTL process was also performed without cosolvent. HTL reactions were performed in 4.1 mL batch minireactors (Swagelok. Solon, Ohio), loaded with 0.3 g of waste biomass (dry basis) and 3 g of water, except where methanol, ethanol, and isopropanol were used as a cosolvent. In those cases, 1.5 g of distilled water and 1.5 g of alcohol were added using biomass/water/solvent ratio of 1:5:5. The reactions were carried out at 300 and 350 °C for 30 min, and the pressure achieved during the reaction was the autogenic pressure. All the HTL reactions were performed in triplicate.

The reactors were heated in a fluidized sand bath (model IFB51 from Techne Inc. Burlington, New Jersey) preheated at the set-point temperature. The heating time to reach set-point temperatures (300 and 350 °C) was 3 min from the introduction of the reactors into the sand bath. At the end of the reaction, reactors were removed from the bath and quenched in an ice-water bath for 5 min. As a result, four phases (biocrude, water-soluble organics, gas phase, and solid residue) were obtained.

The yields of biocrude (*Y*_B_), water-soluble organic products (*Y*_WSO_), and solid residue (*Y*_SR_) were determined on a dry basis using eqs 7–9.^24^ The yield of the gas phase was estimated by mass balance.
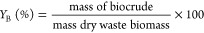
7
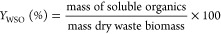
8
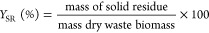
9The biocrude quality was determined by elemental analysis and GC–MS. The high heating value (HHV) for the biocrude and the initial biomass was determined using the results of the elemental analysis (C, H, O, N), according to eq 10(44)
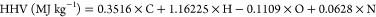
10Finally, the energy recovery (ER) was calculated with eq 11
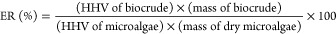
11

## 3 Results and Discussion

### 3.1 Phycocyanin Extraction

PC was extracted from *A. platensis* using [EMIM][EtSO_4_] ionic liquid and sonication according to the procedure described above.^14^ The composition of the extract and the waste biomass is summarized in Table 2.

**Table 2:** Composition of the Extraction Fractions (Dry Weight Basis)

component (wt %)		extract phase	waste biomass
total proteins^b^		87.7 ± 2.0	38.5 ± 1.0
phycobiliproteins	phycocyanin	10.18 ± 0.20	3.60 ± 0.20
	allophycocyanin	2.30 ± 0.09	3.60 ± 0.30
	phycoerythrin	1.41 ± 0.05	1.58 ± 0.10
lipids		3.40^a^	19.8 ± 1.0
total carbohydrates		5.23^a^	35.4 ± 4.0
ash		3.68^a^	6.31 ± 0.20

a Calculated by mass balance.

b Include phycobiliproteins.

Purity is a critical parameter for the commercial use of PC. The extract purity reached a value of 0.54 ± 0.01, corresponding to the lowest commercial grade (grade 1: 0.50–1.50). Therefore, it is mandatory to carry out further downstream processing to obtain PC with a higher purity grade suitable for other uses.

### 3.2 Phycocyanin Purification

PC extract was purified using two approaches (Figure 1): (1) dialysis + precipitation and (2) ATPS + dialysis + precipitation.

#### 3.2.1 Purification Approach 1: Dialysis + Precipitation

The results of the crude extract purification by dialysis + precipitation are shown in Table 3, including the extraction yield, recovery, and purity ratio of PC and purification factor at each step.

**Table 3:** Extraction Yield (*E*_PC_), Recovery, Purity (*P*_PC_), and Purification Factor (PF) of PC for Purification Approach 1 (Dialysis + Precipitation)

step	*E*_PC_ (mg g^–1^)	recovery (%)	*P*_PC_	PF
crude extract of PC	76.6 ± 0.4		0.54 ± 0.01	
dialysis	67.7 ± 0.9	88.4 ± 1.5	0.85 ± 0.03	1.57 ± 0.05
precipitation	48.9 ± 0.9	63.9 ± 0.9	3.5 ± 0.1	6.4 ± 0.2

As seen in Table 3, the extraction yield (*E*_PC_) decreased in the dialysis and the precipitation stages (67.7 ± 0.9 and 48.9 ± 0.9 mg g^–1^, respectively) in comparison with the corresponding value of the crude extract of PC (76.6 ± 0.4 mg g^–1^) because of the extract losses during each purification step. Consequently, the recoveries after the dialysis and precipitation were 88.4 ± 1.5 and 63.9 ± 0.9%, with PC extract losses of 11.6 and 36.2%, respectively.

**Figure 2:**
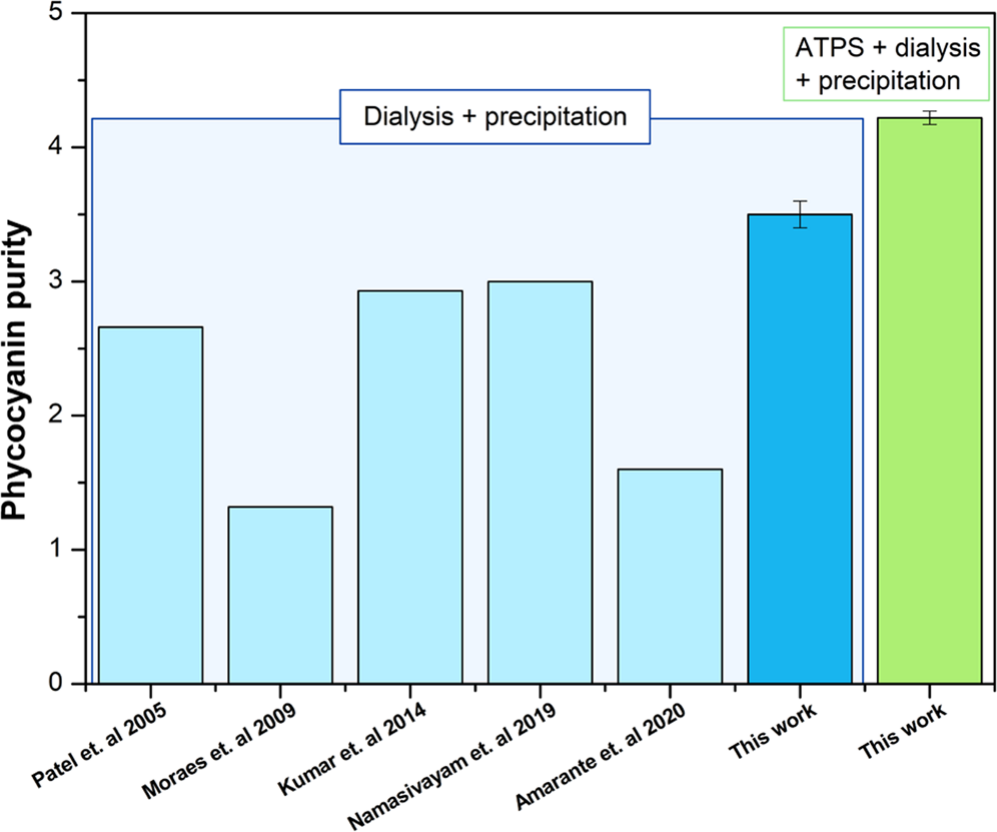
Comparing phycocyanin purity obtained by other researchers and the present work for purification 1 (dialysis + precipitation) and purification 2 (ATPS + dialysis + precipitation) procedures.

However, the purity of PC (*P*_PC_) improved in comparison to that of the PC crude extract (0.54 ± 0.01), achieving values of 0.85 ± 0.03 (dialysis) and 3.5 ± 0.1 (precipitation) within the grades 1 (0.7–1.5) and 3 (2.50–3.50) for the food industry and as a biomarker in biomedical applications, respectively (Figure 2). These results were in line with the purity of PC obtained regarding the crude extract of PC evaluated through the purification factor (PF): 1.57 (dialysis) and 6.4 (precipitation). The latter value means that the purity of PC increased six-fold with respect to that in the crude extract after dialysis + precipitation, showing the suitability of the IL-based extraction process with this purification approach. In addition, the purity of PC obtained through this purification process was significantly higher than the values previously reported by other authors.^35,45−48^ For instance, Kumar et al.^46^ obtained a similar PC purity (2.93) but a lower PC recovery (around 39%). High-purity PC of 3.25 and 3.74, similar to the present work, have been only reported using additional purification stages based on one- or two-step chromatography, respectively.^19^ In these cases, the overall recoveries were as low as 48 and 22%, respectively, which is the main disadvantage of using chromatography techniques to purify PC extract.

#### 3.2.2 Purification Approach 2: ATPS + Dialysis + Precipitation

Table 4 summarizes the downstream processing results of the crude extract following the sequence: ATPS + dialysis + precipitation.

**Table 4:** Extraction Yield (*E*_PC_), Recovery, Purity (*P*_PC_), and Purification Factor (PF) of PC for Purification Approach 2 (ATPS + Dialysis + Precipitation)

step	*E*_PC_ (mg g^–1^)	recovery (%)	*P*_PC_	PF
crude extract of PC	76.6 ± 0.4		0.54 ± 0.01	
top phase (ATPS)	74.0 ± 0.2	96.7 ± 0.3	0.57 ± 0.01	1.07 ± 0.01
dialysis	70.0 ± 0.4	91.3 ± 1.4	2.4 ± 0.3	4.5 ± 0.3
precipitation	46.0 ± 1.0	60.0 ± 1.1	4.22 ± 0.05	7.88 ± 0.06

In this purification approach, the extraction yield (*E*_PC_) decreased slowly after the ATPS (74.0 ± 0.2 mg g^–1^) and the dialysis (70.0 ± 0.4 mg g^–1^) steps regarding the value of the crude extract of PC (76.6 ± 0.4 mg g^–1^). However, the decrease was noticeably higher after the precipitation step (46.0 ± 1.0 mg g^–1^). These results supposed high recoveries using the ATPS system (96.7 ± 0.3%) and the dialysis stage (91.3 ± 1.4%) and a lower recovery with the precipitation stage (60.0 ± 1.1%). Therefore, the final recoveries of both purification approaches (dialysis + precipitation and ATPS + dialysis + precipitation) were very similar (63.9 ± 0.9 and 60.0 ± 1.1%, respectively). In addition, the recoveries of PC were superior to the ones obtained in other works where additional steps were used, such as gel filtration chromatography and ion-exchange chromatography.^19,46^

The purity of PC (*P*_PC_) after the ATPS step was only slightly higher (0.57 ± 0.01) than the purity of the crude extract (0.54 ± 0.01) in this downstream process. Consequently, the purification factor with ATPS was only 1.07 ± 0.01. These results can be explained since the ATPS selectively extract proteins in the top phase, favoring PC isolation from the rest of the proteins in the subsequent dialysis and precipitation stages. To check this hypothesis, both the top and bottom phases obtained in the ATPS stage were analyzed, showing that 89.4 wt % of total proteins were partitioned in the top phase, whereas 38.6 wt % of the remaining carbohydrates were accumulated in the bottom phase. Thus, it is demonstrated that the ATPS system was helpful in the separation of carbohydrates from PC.

Conversely, the purity value increased to 2.4 ± 0.3 and 4.22 ± 0.05 and the purification factor to 4.5 ± 0.3 and 7.88 ± 0.06 after dialysis and precipitation steps, respectively. Thus, the PC purity obtained with this purification process (ATPS + dialysis + precipitation) achieved the analytical grade of purity (>4) for pharmaceutical and nutraceutical purposes, being higher than the one (3.5) obtained in the previous purification approach (dialysis + precipitation) (Figure 2).

### 3.3 Hydrothermal Liquefaction of Waste Biomass

**Figure 3:**
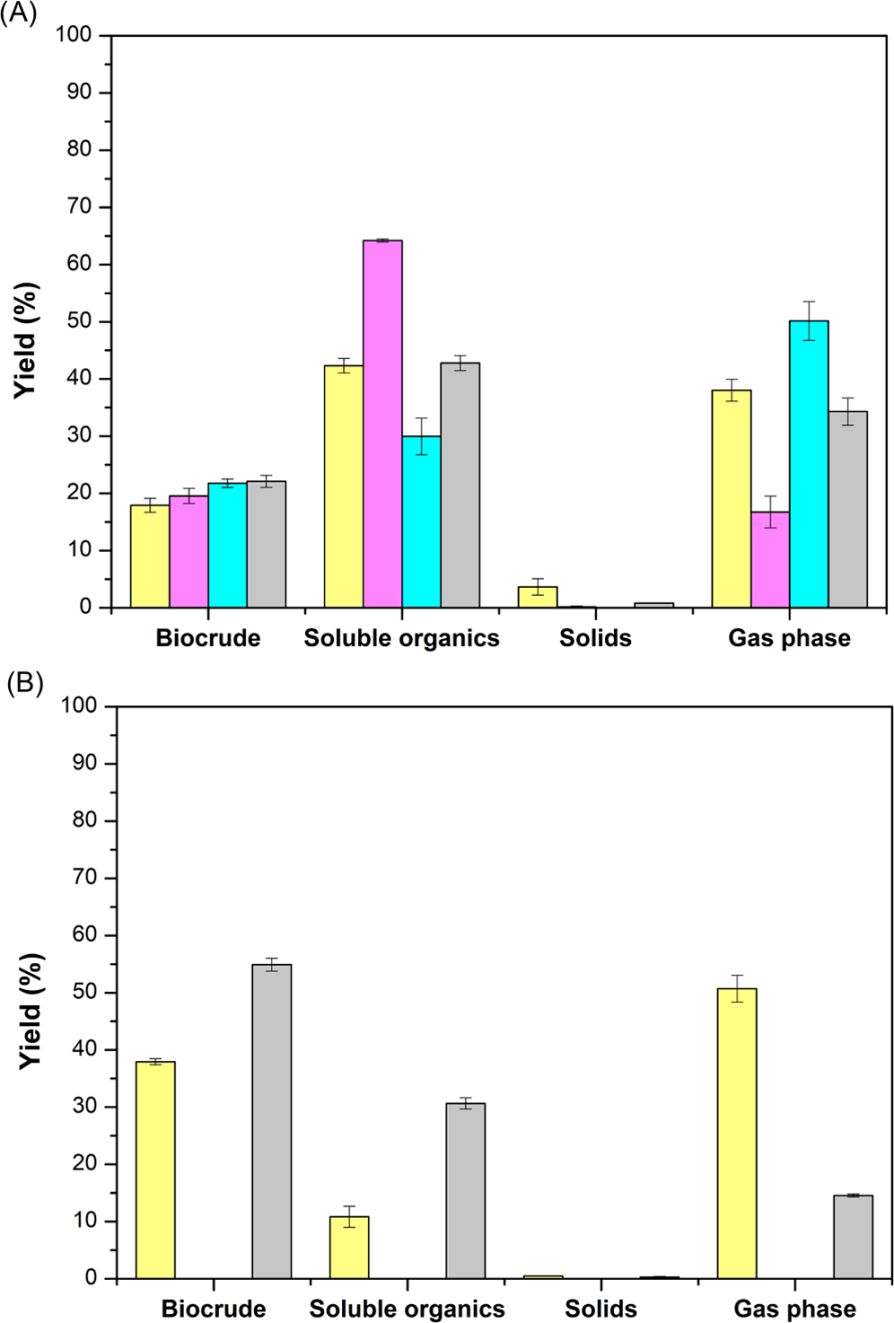
HTL product yields from waste biomass at 300 °C (A) and 350 °C (B) with different solvents: methanol (pink), ethanol (cyan), and isopropanol (gray) and solventless (yellow). Error bars correspond to the standard deviation of the three replicates.

HTL was carried out using the wet waste biomass (obtained after the extraction process) at different temperatures (300 and 350 °C) using different cosolvents (methanol, ethanol, and isopropanol). The yield of the different fractions from the HTL process at 300 and 350 °C (biocrude, water-soluble organics, gas phase, and solid residue) is shown in Figure 3.

At both temperatures (300 and 350 °C), the highest biocrude yield was reached when the wet waste biomass was treated with cosolvents (alcohols) with respect to that treated without solvent (Figure 3). This result agrees with previous studies,^25,44−46^ showing that alcohols improved the yield and quality of biocrude. The use of alcohols presents several advantages over only water during the HTL process since they are better hydrogen donors to stabilize free radicals, improve the solubility of organic compounds into the biocrude oil phase, and have high reactivity with acidic components to form esters.

Among the cosolvent tested, at 300 °C, ethanol and isopropanol are the cosolvents showing higher biocrude yield values (21.8 ± 0.8 and 22.1 ± 1.1%, respectively). At 350 °C, only isopropanol was used as solvent since the pressure inside the reactor exceeded the limit when methanol and ethanol were used, as might be expected by examining the phase equilibrium diagrams for methanol and ethanol. For all the systems studied in the present work, the biocrude yield increased with temperature, showing a similar pattern to that reported in the literature for raw microalgal biomass.^49,50^ However, the biocrude yield using isopropanol was remarkably higher (54.9 ± 1.1%).

Table 5 summarizes the elemental composition and the higher heating value (HHV) of microalgal waste after the PC extraction and the biocrude obtained at 350 °C by HTL.

**Table 5:** Elemental Analysis (wt %, Dry Basis), Higher Heating Value (HHV), and Energy Recovery (ER) of the Waste Biomass and Biocrude after the Hydrothermal Liquefaction (HTL) Process at 350 °C for 30 min

biomass/biocrude	C (%)	H (%)	N (%)	S (%)	O (%)	HHV (MJ kg^–1^)	ER (%)
waste biomass	48.8 ± 0.1	7.20 ± 0.13	9.20 ± 0.03	0.24 ± 0.04	22.4 ± 0.5	26.9 ± 0.2	
biocrude from wet waste biomass	74.4 ± 0.3	9.70 ± 0.02	6.5 ± 0.1	0.34 ± 0.03	9.04 ± 0.04	37.6 ± 0.2	55.9 ± 0.4
biocrude from wet waste biomass + isopropanol	76.3 ± 0.2	10.4 ± 0.1	5.2 ± 0.2	0.60 ± 0.02	7.5 ± 0.2	39.7 ± 0.2	78.4 ± 0.6

The elemental composition of the biomass did not change significantly with the PC extraction process, being very similar. Therefore, for the *A. platensis,* initial biomass and the waste biomass were obtained in the extraction process (Tables 1 and 5).

The carbon and hydrogen contents in biocrude oils, which contribute to higher HHV, were 74.4 ± 0.3 and 9.70 ± 0.02 wt % for conventional HTL and 76.3 ± 0.2 and 10.4 ± 0.1 wt % for HTL with isopropanol, respectively. These values were significantly higher than the corresponding ones in the waste biomass (48.8 ± 0.1 and 7.20 ± 0.13 wt %, respectively).

Conversely, the oxygen content in both biocrudes was significantly lower than this element content in the starting waste biomass (22.4 ± 0.5 wt %). The reduction of oxygen leads to an increase in the HHV of the biocrudes. These results indicate the presence of decarboxylation reactions during the HTL process in both cases. In particular, the oxygen amount in the biocrude was lower when isopropanol was used as a cosolvent (7.5 ± 0.2 wt %) compared to the solventless process (9.04 ± 0.04 wt %). This fact may be due to the ability of isopropanol to act as an efficient hydrogen donor solvent, which enhances dehydration reactions.^51^

The nitrogen content of the biocrude obtained from the HTL without isopropanol was 6.5 ± 0.1 wt %, slightly higher than that from the HTL with this alcohol (5.2 ± 0.2 wt %). Therefore, a decrease was observed in this heteroatom content in the biocrudes of both HTL with respect to the residual biomass (9.20 ± 0.03 wt %), which indicates the presence of hydrolysis and cracking reactions during HTL breaking the macromolecules (proteins and lipids) to give nitrogen compounds soluble in the aqueous layer.

Despite the significant reduction of oxygen and nitrogen observed in the biocrudes, they cannot still be used as transport fuels. Therefore, a hydrotreating stage is required to reduce their contents and, thus, improve their chemical composition to fulfill the specifications of these heteroatoms in the commercial standards.

According to the elemental analysis, the calculated HHV of the biocrudes were significantly higher (37.6 ± 0.2 and 39.7 ± 0.2 MJ kg^–1^ in the absence and presence of isopropanol, respectively) than the HHV in the initial waste biomass (26.9 ± 0.2 MJ kg^–1^) due to the observed decrease in the oxygen content as well as the increase of the carbon and hydrogen contents in the biocrudes.

The energy recovered (ER) in the biocrude obtained from waste biomass in the presence of only water was lower (55.9 ± 0.4%) than the energy recovered for waste biomass using isopropanol (78.4 ± 0.6%), which is in agreement with the literature,^52^ because of the lower biocrude yield in the conventional HTL process.

**Figure 4:**
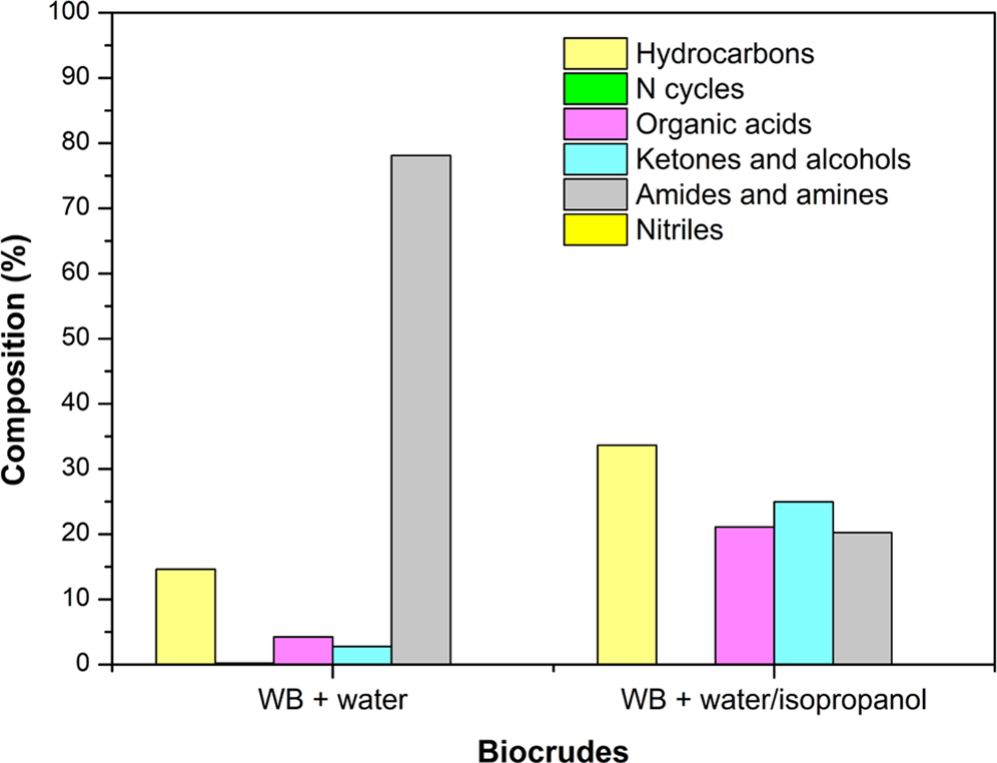
GC–MS analysis results of the biocrude obtained from *A. platensis* waste biomass (WB) by HTL at 350 °C.

Figure 4 shows the GC–MS analysis of the biocrudes obtained from HTL at 350 °C from *A. platensis* waste biomass, classified by the type of compound detected according to the main functional group in the molecule.

The major compounds of biocrudes were amides, amines, ketones, alcohols, hydrocarbons, and organic acids. Both amides and amines are produced from the hydrolysis of proteins, whereas organic acids are mainly generated during the hydrolysis of lipids and the hydrolysis and deamination of proteins.^53^ The increase in organic acids observed when isopropanol was used means that the lipids can be extracted more effectively in the presence of this solvent and hydrolyzed to form organic acids. In addition, the amide content in the biocrude decreased in the HTL using isopropanol since this solvent promotes the deamination reactions of proteins. Remarkably, the hydrocarbon content in the biocrude increased considerably (two-fold) when isopropanol was used (33.7 ± 0.8%) compared to that when the HTL process was carried out without this solvent (14.6 ± 0.4%).

## 4 Conclusions

An innovative biorefinery to extract and purify PC from *A. platensis* along with the production of biocrude by HTL was studied. The PC extraction was carried out using the IL [Emim][EtSO_4_]. The extract was then purified using two downstream processings: (1) dialysis + precipitation and (2) ATPS + dialysis + precipitation. The purification approach 2 produced high recoveries and analytical-grade purity for pharmaceutical and nutraceutical applications. The HTL of the waste biomass in the presence of isopropanol at 350 °C enhances the biocrude yield and composition with high carbon and hydrogen contents, low oxygen and nitrogen amounts, high content of hydrocarbons, and therefore high HHV.

## Supporting Information Available

Construction of binodal curve and model fitting; determination of tie-lines (PDF)
