# Supplementary material for: Novel Biorefinery Approach for Phycocyanin Extraction and Purification and Biocrude Production from Arthrospira platensis
Source: Ind Eng Chem Res. 2023 Mar 8;62(12):5190–8. doi: 10.1021/acs.iecr.2c03683 (PMC10064637; doi:10.1021/acs.iecr.2c03683)
Supplement: Supplementary file 1 — ie2c03683_si_001.pdf [file ie2c03683_si_001.pdf]

A novel biorefinery approach for phycocyanin extraction and purification and biocrude production from *Arthrospira platensis*

*Jennifer Sánchez-Laso[1], Juan J. Espada[2], Rosalía Rodríguez[2] Gemma Vicente[2], Luis Fernando Bautista[1]**

[1] Department of Chemical and Environmental Technology. ESCET, Universidad Rey Juan Carlos, 28933, Móstoles, Madrid, Spain.

[2] Department of Chemical, Energy and Mechanical Technology. ESCET, Universidad Rey Juan Carlos, 28933, Móstoles, Madrid, Spain.

SUPPLEMENTARY MATERIAL

EXPERIMENTAL SECTION

1.1. Construction of binodal curve and model fitting

The biphasic equilibrium of the ATPS system is described by the binodal curve which shows the concentration of the ionic liquid (i.e., [Emim][EtSO$_4$]) in the upper phase and the concentration of the salt (K_2HPO_4) in the lower phase (Figure S1).

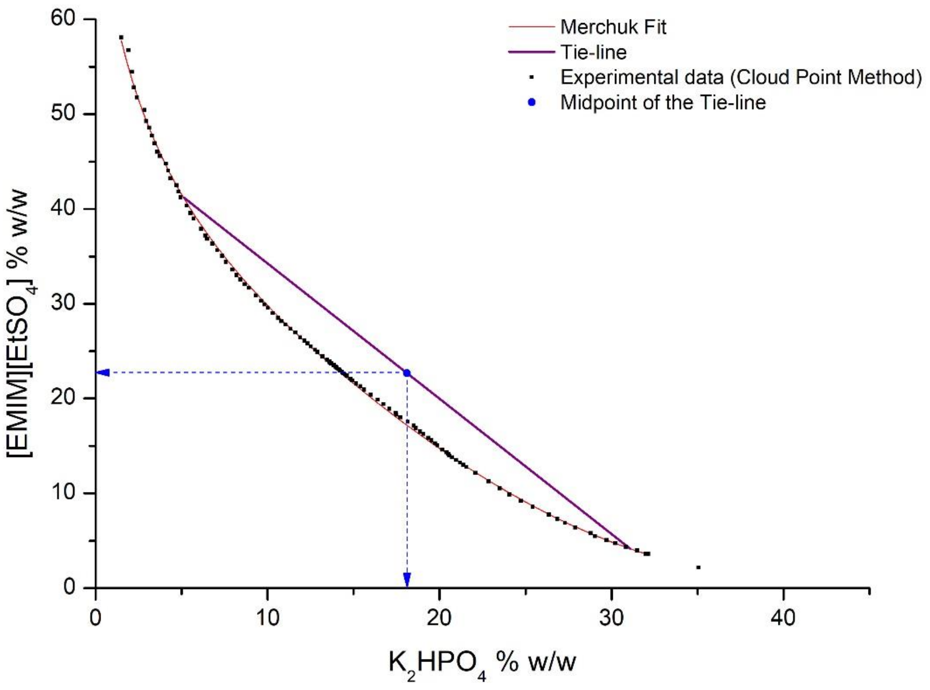

Figure S1: binodal curve for the ATPS system formed by the ionic liquid [Emim][$EtSO_4$] and the inorganic salt K_2HPO_4 in aqueous solution at 25ºC.

The binodal curves were determined by the Cloud Point method [1,2]. The ionic liquid (IL) [Emim][$EtSO_4$] solution with known mass fraction was added to a glass vessel. Then, a salt solution of known mass fraction was added dropwise to the vessel until the mixture became turbid or cloudy (biphasic region). The weight of salt solution was stored.

Then, pure water was added to the system dropwise to the vessel to get a transparent one-phase system (monophasic region), and the procedure was repeated until all points in the binodal curve were obtained.

The construction of binodal curves was made at 25ºC and atmospheric pressure, and all the additions were made under constant and controlled stirring. The composition of the

mixture for each point on the binodal curve was calculated by mass using an analytical balance.

The experimental equilibrium curve was fitted to the Merchuk equation [3]:

$$Y = A \cdot exp[(B \cdot X^{0.5}) - (C \cdot X^{3})] \qquad (Eq. 1)$$

Where Y and X represent the IL and salt mass fraction percentages, respectively, and A, B, and C are constants obtained by the regression of the experimental binodal data.

1.2. Determination of tie-lines

Once the fitting to Merchuk equation was made, the tie-lines (TLs) were calculated. A tie-line is a line that has a specific composition, which is identical along the tie-line; however, it has different mass and volume relationships in the coexisting phases.

The tie-lines were determined by a gravimetric method initially proposed by Merchuk et al [3]. In this method, a point in the biphasic region (IL+salt+water) of the binodal curve was chosen randomly and gravimetrically prepared. An ATPS was formed with the corresponding amounts of ionic liquid, salt and water of the chosen point and the mixture was stirred in a vortex mixer for 30 s and left for at least half an hour in a bath at 25°C to reach the complete separation and equilibration of the coexisting phases.

After complete separation of the phases, both top and bottom phases were weighed.

For the determination of TLs the following system of five equations with five unknowns was used [3,4]:

$$Y_T = A \cdot exp\left[\left(B \cdot {X_T}^{0.5}\right) - \left(C \cdot {X_T}^{3}\right)\right] \qquad (Eq. 2)$$

$$Y_B = A \cdot exp\left[\left(B \cdot {X_B}^{0.5}\right) - \left(C \cdot {X_B}^{3}\right)\right] \qquad (Eq. 3)$$

$$Y_T = \frac{Y_M}{\propto} - \frac{1-\propto}{\propto} \cdot Y_B \qquad (Eq. 4)$$

$$X_T = \frac{X_M}{\propto} - \frac{1-\propto}{\propto} \cdot X_B \qquad (Eq. 5)$$

Where T, B and M, designate the top phase, the bottom phase, and the mixture, respectively. X and Y represent the weight fraction percentage of the salt and of the ionic liquid, respectively; and α is the ratio between the top weight and the total weight of the mixture [5]:

$$\propto = \frac{w_{Top}}{w_{total}} \qquad (Eq. 6)$$

The solution of this system yields the concentration (wt%.) of the IL and salt in the top and bottom phases and, therefore, the TLs can be represented.

REFERENCES

(1) Ventura, S. P. M.; Neves, C. M. S. S.; Freire, M. G.; Marrucho, I. M.; Oliveira, J.; Coutinho, J. A. P. Evaluation of Anion Influence on the Formation and Extraction Capacity of Ionic-Liquid-Based Aqueous Biphasic Systems. *J. Phys. Chem. B* **2009**, *113* (27), 9304–9310. https://doi.org/10.1021/jp903286d.

(2) Domínguez-Pérez, M.; Tomé, L. I. N.; Freire, M. G.; Marrucho, I. M.; Cabeza, O.; Coutinho, J. A. P. (Extraction of Biomolecules Using) Aqueous Biphasic Systems Formed by Ionic Liquids and Aminoacids. *Sep. Purif. Technol.* **2010**, *72* (1), 85–91. https://doi.org/10.1016/j.seppur.2010.01.008.

(3) Merchuk, J. C.; Andrews, B. A.; Asenjo, J. A. Aqueous Two-Phase Systems for Protein Separation Studies on Phase Inversion. *J. Solution Chem.* **1998**, *711* (12), 285–293. https://doi.org/10.1007/s10953-016-0547-x.

(4) Freire, M. G.; Cláudio, A. F. M.; Araújo, J. M. M.; Coutinho, J. A. P.; Marrucho, I. M.; Canongia Lopes, J. N.; Rebelo, L. P. N. Aqueous Biphasic Systems: A Boost Brought about by Using Ionic Liquids. *Chem. Soc. Rev.* **2012**, *41* (14), 4966–4995. https://doi.org/10.1039/c2cs35151j.

(5) Ferreira, A. M.; Coutinho, J. A. P.; Fernandes, A. M.; Freire, M. G. Complete Removal of Textile Dyes from Aqueous Media Using Ionic-Liquid-Based Aqueous Two-Phase Systems. *Sep. Purif. Technol.* **2014**, *128*, 58–66. https://doi.org/10.1016/j.seppur.2014.02.036.
